# PknB remains an essential and a conserved target for drug development in susceptible and MDR strains of *M. Tuberculosis*

**DOI:** 10.1186/s12941-017-0234-9

**Published:** 2017-08-18

**Authors:** Anamika Gupta, Sudhir K. Pal, Divya Pandey, Najneen A. Fakir, Sunita Rathod, Dhiraj Sinha, S. SivaKumar, Pallavi Sinha, Mycal Periera, Shilpa Balgam, Gomathi Sekar, K. R. UmaDevi, Shampa Anupurba, Vijay Nema

**Affiliations:** 1 Department of Molecular Biology, National AIDS Research Institute, 73 G MIDC, Bhosari, Pune 411 026 India; 2 National Institute for Research in Tuberculosis, No.1, Mayor Sathiyamoorthy Road, Chetpet, Chennai, 600 031 India; 3 Department of Microbiology, Institute of Medical Sciences, Banaras Hindu University, Varanasi, 221 005 India; 4 Department of Microbiology and Clinical Pathology, National AIDS Research Institute, 73 G MIDC Bhosari, Pune, 411026 India; 5 Intermediate Reference Laboratory State TB Training and Demonstration Centre (STDC) Pune, Pune, 411027 India; 6 Department of Bioinformatics, IIDS, Nehru Science Center, University of Allahabad, Allahabad, 211002 India

**Keywords:** PknB, *M. tuberculosis*, Conservation, Multidrug resistant, Mutation, Sequencing

## Abstract

**Background:**

The *Mycobacterium tuberculosis* (*M.tb*) protein kinase B (PknB) which is now proved to be essential for the growth and survival of *M.tb*, is a transmembrane protein with a potential to be a good drug target. However it is not known if this target remains conserved in otherwise resistant isolates from clinical origin. The present study describes the conservation analysis of sequences covering the inhibitor binding domain of PknB to assess if it remains conserved in susceptible and resistant clinical strains of mycobacteria picked from three different geographical areas of India.

**Methods:**

A total of 116 isolates from North, South and West India were used in the study with a variable profile of their susceptibilities towards streptomycin, isoniazid, rifampicin, ethambutol and ofloxacin. Isolates were also spoligotyped in order to find if the conservation pattern of *pknB* gene remain consistent or differ with different spoligotypes. The impact of variation as found in the study was analyzed using Molecular dynamics simulations.

**Results:**

The sequencing results with 115/116 isolates revealed the conserved nature of *pknB* sequences irrespective of their susceptibility status and spoligotypes. The only variation found was in one strains wherein *pnkB* sequence had G to A mutation at 664 position translating into a change of amino acid, Valine to Isoleucine. After analyzing the impact of this sequence variation using Molecular dynamics simulations, it was observed that the variation is causing no significant change in protein structure or the inhibitor binding.

**Conclusions:**

Hence, the study endorses that PknB is an ideal target for drug development and there is no pre-existing or induced resistance with respect to the sequences involved in inhibitor binding. Also if the mutation that we are reporting for the first time is found again in subsequent work, it should be checked with phenotypic profile before drawing the conclusion that it would affect the activity in any way. Bioinformatics analysis in our study says that it has no significant effect on the binding and hence the activity of the protein.

## Background

The emergence of multi-drug and extensively drug resistant strains (MDR & XDR) of *Mycobacterium tuberculosis (M.tb)* has highlighted the need for new drugs to treat tuberculosis (TB). As the targets of current drugs are modified by the resistant organisms, newer targets which remain conserved in clinical isolates remain the choice for drug development. Serine/threonine protein kinases (STPK) remain one such target that plays important roles in mycobacterial cellular processes, including cell division, cell wall synthesis, cell metabolism, etc. [1]. Protein kinases A and B, encoded by *pknA* and *pknB*, respectively, are part of the operon carrying cistrons coding for protein phosphatase *pstP*, *rodA* (involved in cell shape control), and *pbpA* (involved in peptidoglycan synthesis). This locus is found near the origin of replication throughout the *Mycobacterium* genus [2]. Both these kinases have been found to be essential based on transposon-insertion experiments [3]. A role for PknB as a replication switch in response to hypoxia has been proposed with its essential role in reactivation of cells from the hypoxic state [4]. Hence, the Ser/Thr protein kinase PknB is proved to be essential for sustaining mycobacterial growth and this support the idea of the development of protein kinase inhibitors as new potential anti-tuberculosis drugs. However, to the best of our knowledge, not much work has been done for exploring the conservation of these genes in clinical isolates. In the present study, we probed the conservation pattern of the target region by sequencing this gene in clinical strains of *M.tb* and verified the same by deciphering the role of mutation in the drug-protein interactions in the PknB protein of *M.tb* using bioinformatics analysis. These isolates were either susceptible or resistant to the drugs being used currently and had different spoligotypes.

## Methods

### Ethics permission

The study was approved by the institutional ethics committee and all the experiments were undertaken by the trained staff at BSL3 facility.

### Isolates used

A total of 116 *Mycobacterium tuberculosis* isolates were obtained from three different geographical regions of India, viz., Pune (Western India), Varanasi (Northern India) and Chennai (Southern India). Also, two sets of isolates were obtained from Pune wherein set 1 was an older collection of the isolates (2006–2007) and set 2 was a recent collection (2012–2013). Isolates from Chennai and Varanasi were collected during 2013–2014 (Table 1).

**Table 1:** Demographic and laboratory profile of the isolates used

Sr. no.	Study no.	HIV	Age	Sex	Collection date	Drug susceptibility profile (MGIT)	Spoligotype	Mutations in *pknB* gene
1	N1	N	35	F	6/3/2006	Susceptible	T1-53	Nil
2	N2	N	50	F	17/03/2006	Susceptible	Orphan	Nil
3	N3	N	30	M	24/03/2006	Susceptible	EAI5-355	Nil
4	N4	N	50	M	27/03/2006	Susceptible	EAI3_IND-11	Nil
5	N5	N	22	F	29/03/2006	Susceptible	CAS1_DELHI-26	Nil
6	N6	N	38	M	28/04/2006	Susceptible	CAS1_DELHI-26	Nil
7	N7	N	55	F	8/5/2006	Susceptible	CAS-486	Nil
8	N8	N	29	F	15/06/2006	Susceptible	CAS-357	Nil
9	N9	N	23	M	16/06/2006	Susceptible	MANU2-1634	Nil
10	N10	N	23	M	19/06/2006	Susceptible	EAI5-126	Nil
11	N11	N	40	F	27/06/2006	Susceptible	CAS1_DELHI-427	Nil
12	N12	N	28	M	29/06/2006	Susceptible	T3-37	Nil
13	N13	N	40	M	29/06/2006	Susceptible	CAS1_DELHI-25	Nil
14	N14	N	24	M	10/10/2006	Susceptible	CAS1_DELHI-26	Nil
15	N15	N	22	M	20/10/2006	Susceptible	EAI1_SOM-48	Nil
16	N16	N	25	F	11/5/2006	S,H	T2-52	**664 (GTC-ATC)**
17	N17	N	32	M	5/7/2006	S,H,R	Orphan	Nil
18	N18	N	30	M	12/9/2006	S,H,R	CAS2-288	Nil
19	N19	N	21	M	22/09/2006	S,H	T3-37	Nil
20	N20	N	27	M	27/09/2006	S	Orphan	Nil
21	N21	N	42	M	31/10/2006	S	EAI5-126	Nil
22	N22	N	26	M	15/01/2007	S,H	Orphan	Nil
23	N23	N	21	M	18/01/2007	H	H3-99	Nil
24	N24	N	38	M	22/01/2007	S,H,E	CAS1_DELHI-26	Nil
25	N25	N	45	F	31/01/2007	H	CAS1_DELHI-26	Nil
26	N26	P	32	M	7/2/2007	S,H,R	T2-1077	Nil
27	N27	P	40	M	7/3/2007	S,H	CAS2-288	Nil
28	N28	N	50	M	8/3/2007	S	H3-99	Nil
29	N29	P	35	M	8/3/2007	H	Orphan	Nil
30	S1	NT	35	M	27/03/2013	Susceptible	CAS1_DELHI-26	Nil
31	S2	NT	27	M	3/4/2013	Susceptible	T1-53	Nil
32	S3	NT	50	M	3/4/2013	Susceptible	Orphan	Nil
33	S4	NT	24	M	3/4/2013	H,R,E,OFX	Orphan	Nil
34	S5	NT	38	M	4/4/2013	S,H,R,E	BEIJING-1	Nil
35	S6	NT	45	M	5/4/2013	R,H	T1-53	Nil
36	S7	NT	31	M	8/4/2013	Susceptible	T1-53	Nil
37	S8	NT	50	M	3/4/2013	R,H	CAS1_DELHI-1789	Nil
38	S9	NT	35	M	8/4/2013	Susceptible	Orphan	Nil
39	S10	NA	55	M	8/4/2013	OFX	MANU1-100	Nil
40	S11	NA	NA	NA	NA	S,H,E,OFX	BEIJING-1	Nil
41	S12	P	40	F	2012–2013	Susceptible	EAI3_IND-11	Nil
42	S13	P	NA	NA	2012–2013	Susceptible	CAS1_DELHI-26	Nil
43	S14	P	35	M	2012–2013	Susceptible	CAS1_DELHI-26	Nil
44	S15	P	48	F	2012–2013	H	CAS1_DELHI-26	Nil
45	S16	P	26	M	2012–2013	H,R	CAS1_DELHI-26	Nil
46	S17	P	30	F	2012–2013	H	CAS1_DELHI-26	Nil
47	S18	P	31	M	2012–2013	H,R,OFX	Orphan	Nil
48	S19	P	36	M	2012–2013	H	Orphan	Nil
49	S20	P	23	M	2012–2013	H	Orphan	Nil
50	C1	NT	NA	NA	2013	R,H, OFX	T5-1268	Nil
51	C2	NT	NA	NA	2013	R,H	EAI3_IND-11	Nil
52	C3	NT	NA	NA	2013	R,H	BEIJING-1	Nil
53	C4	NT	NA	NA	2013	R,H,OFX	Orphan	Nil
54	C5	NT	NA	NA	2013	R,H	Orphan	Nil
55	C6	NT	NA	NA	2013	R,H,OFX	CAS1_DELHI-381	Nil
56	C7	NT	NA	NA	2013	R,H	T1–T2-78	Nil
57	C8	NT	NA	NA	2013	R,H,OFX	T2-52	Nil
58	C9	NT	NA	NA	2013	R,H,OFX	Orphan	Nil
59	C10	NT	NA	NA	2013	R,H,OFX	Orphan	Nil
60	C11	NT	NA	NA	2013	R,H	T1-53	Nil
61	C12	NT	NA	NA	2013	R,H	Orphan	Nil
62	C13	NT	NA	NA	2013	S,H,R,E,OFX	Orphan	Nil
63	C14	NT	NA	NA	2013	Susceptible	CAS1_DELHI-26	Nil
64	C15	NT	NA	NA	2013	S,H,E	T2-52	Nil
65	C16	NT	NA	NA	2013	S,H,R,E	Bejing-1	Nil
66	C17	NT	NA	NA	2013	S,E	CAS1_DELHI-26	Nil
67	C18	NT	NA	NA	2013	S,E	Orphan	Nil
68	C19	NT	NA	NA	2013	S	Orphan	Nil
69	C20	NT	NA	NA	2013	S	Orphan	Nil
70	C21	NT	NA	NA	2013	Susceptible	EA15-934	Nil
71	C22	NT	NA	NA	2013	S,H,R,E	EA15-934	Nil
72	C23	NT	NA	NA	2013	Susceptible	EAI3_IND-11	Nil
73	C24	NT	NA	NA	2013	Susceptible	Orphan	Nil
74	C25	NT	NA	NA	2013	Susceptible	Bejing-1	Nil
75	C26	NT	NA	NA	2013	S,H,R,E		Nil
76	C27	NT	NA	NA	2013	Susceptible	T1-804	Nil
77	C28	NT	NA	NA	2013	H	EAI3_IND	Nil
78	C29	NT	NA	NA	2013	Susceptible	EAI3_IND	Nil
79	C30	NT	NA	NA	2013	S,H,R,E,OFX	Orphan	Nil
80	C31	NT	NA	NA	2013	Susceptible	Orphan	Nil
81	C32	NT	NA	NA	2013	Susceptible	CAS1_DELHI	Nil
82	C33	NT	NA	NA	2013	S,H, OFX	Bejing-1	Nil
83	C34	NT	NA	NA	2014	Susceptible	EAI3_IND-11	Nil
84	C35	NT	NA	NA	2014	S,H,OFX	Orphan	Nil
85	C36	NT	NA	NA	2014	OF	U-1391	Nil
86	C37	NT	NA	NA	2014	Susceptible	EAI3_IND-11	Nil
87	C38	NT	NA	NA	2014	Susceptible	EAI3_IND-11	Nil
88	C39	NT	NA	NA	2014	R	EAI3_IND-11	Nil
89	C40	NT	NA	NA	2014	Susceptible	Orphan	Nil
90	C41	NT	NA	NA	2014	Susceptible	EAI3_IND-11	Nil
91	B1	N	18	F	2013–2014	S,H,R,E,OFX	BEIJING-1	Nil
92	B2	N	20	M	2013–2014	S,H,R,E,OFX	H1-47	Nil
93	B3	P	25	F	2013–2014	S	LAM2–LAM4-194	Nil
94	B4	N	25	M	2013–2014	S,H,R,E, OFX	LAM-9	Nil
95	B5	N	40	M	2013–2014	S,H	CAS2-288	Nil
96	B6	N	25	M	2013–2014	Susceptible	Orphan	Nil
97	B7	N	47	M	2013–2014	S,H,R,E	Orphan	Nil
98	B8	N	30	M	2013–2014	S,H,R,E	BEIJING-1	Nil
99	B9	N	20	M	2013–2014	S,H,R,E,OFX	T1-53	Nil
100	B10	N	21	M	2013–2014	H,R	Orphan	Nil
101	B11	N	45	M	2013–2014	H,R,OFX	BEIJING-1	Nil
102	B12	N	23	M	2013–2014	S,H	T1-53	Nil
103	B13	N	23	M	2013–2014	Susceptible	EAI1_SOM-235	Nil
104	B14	N	22	F	2013–2014	S,H,R,OFX	CAS1_DELHI	Nil
105	B15	N	22	F	2013–2014	S,H,R,E,OFX	CAS1_DELHI	Nil
106	B16	N	70	M	2013–2014	H,R,OFX	Orphan	Nil
107	B17	N	9	F	2013–2014	H,R,E,OFX	BEIJING-1	Nil
108	B18	N	19	M	2013–2014	H,R,E	Orphan	Nil
109	B19	N	25	F	2013–2014	H	T1-53	Nil
110	B20	P	45	F	2013–2014	Susceptible	BEIJING-1	Nil
111	B21	N	30	F	2013–2014	S,H,R,E,OFX	BEIJING-1	Nil
112	B22	N	18	F	2013–2014	S,H,R,E,OFX	BEIJING-1	Nil
113	B23	N	46	F	2013–2014	S	Orphan	Nil
114	B24	N	50	M	2013–2014	H,R,OFX	BEIJING-1	Nil
115	B25	N	NA	NA	2013–2014	OFX	T1-1567	Nil
116	B26	N	NA	NA	2013–2014	H,S	U-172	Nil

*N1–N29* isolates obtained from NARI, Pune; *S1–S20* isolates obtained from STDC, Pune, Maharashtra; *C1–C40* isolates obtained from NIRT, Chennai, Tamil Nadu; *B1–B26* isolates obtained from BHU, Varanasi, Uttar Pradesh

*NIRT* National Institute for Research in Tuberculosis, *BHU* Banaras Hindu University, *STDC* State TB Training and Demonstration Centre, *S* resistant to streptomycin, *H* resistant to isoniazid, *R* resistant to rifampicin, *E* resistant to ethambutol, *OFX* resistant to ofloxacin, *N* negative, *NT* not tested, *NA* information not available

### Sub-culturing

Upon arrival to our laboratory, the isolates were sub-cultured on Löwenstein-Jensen (LJ) medium and incubated till sufficient growth was indicated by typical non-pigmented, rough, dry colonies.

### Drug susceptibility testing

Isolates were subjected to drug susceptibility testing on BACTEC™ MGIT™ 960 Mycobacterial Detection System (BD, NJ, USA) using SIRE kit for the susceptibility testing against streptomycin (S), isoniazid (I), rifampin (R) and ethambutol (E). For susceptibility testing against ofloxacin, blank tubes of medium were used wherein the final concentration of ofloxacin was kept to 2 μg/ml as per the CLSI standards using filtered stock solution of the drug. The inoculum was prepared from fresh LJ medium by following the methods used by Devasia and co-workers [5]. A growth control containing no antibiotic and a sterile control without inoculation were also used with every set of experiment.

### DNA isolation and amplification of *pknB* gene

Genomic DNA isolation was done from freshly sub-cultured isolates using a cetyltrimethyl ammonium bromide (CTAB) based modified method as reported by earlier workers [6, 7]. The DNA so obtained was quantitated using NanoDrop 1000 spectrophotometer (Thermo, USA) and purity and integrity were checked on 0.8% agarose gel after electrophoresis. A length of 876 bp covering the cytosolic portion of the protein was amplified using forward primer as AATGACCACCCCTTCCCA and reverse primer as TCGGCA TCGGTGAGCACTTT designed with *Mycobacterium tuberculosis* H37Rv (NC_000962;16594-17469) as a template. The amplification reaction was done using KAPA HiFi DNA Polymerase (KAPA Biosystems, USA) using manufacturer’s instructions and annealing at 65 °C.

### Spoligotyping

Spoligotyping was performed using the standard method at National Institute for Research in Tuberculosis, Chennai [8]. In brief, the spacers between the direct repeats in the target region were amplified by using a primer set, primer Dra 5′-CC AAG AGG GGA CGG AAA C-3′ and biotinylated primer Drb 5′-GGT TTT GGG TCT GAC GAC-3′. The polymerase chain reaction (PCR) products were then hybridized to a Biodyne C membrane (Isogen Bioscience). This membrane contains immobilized synthetic oligomeric spacer sequences derived from the direct-repeat region of *M. tuberculosis* H37Rv and *Mycobacterium bovis* BCG. Hybridized DNA was detected using an enhanced chemiluminescence kit (Amersham International), with exposure to X-ray film producing a pattern or profile reminiscent of a bar code. These were matched with the SITVIT web database to get spoligotypes matched with available profiles (http://www.pasteur-guadeloupe.fr:8081/SITVIT_ONLINE/).

### Sequencing and sequence analysis

For sequencing of the amplified gene of pknB, same primers which were used for amplification were employed. The sequencing of plasmid DNA was carried out using Big Dye Terminator v. 3.1 cycle sequencing kit and an ABI 3730xl DNA analyzer, according to the manufacturer’s protocol. Nucleotide sequence analysis and curing were done using Applied Biosystem SeqScape v.2.5. The cured sequences were compared for sequence similarities using Rv0014c from NC_000962 as a template in SeqScape software.

### Bioinformatics analysis

Based on sequencing of the pknB gene, the sequence that showed mutation was taken for bioinformatics analysis to access the impact of the change. Molecular dynamics simulations (MD) tool have potential to provide detailed information on the fluctuations and conformational changes of proteins and hence was used here. MD-simulations were performed to the docked complex to evaluate the stability, conformational changes and getting insights into the natural dynamic in solutions. MD-simulations were performed using GROMACS version-4.6.4 [9–11], with the AMBER99SB force field [12]. The ligand was parameterized by applying the standard RESP procedure using Antechamber [13], where charges for free ligand were derived from HF/6-31G* calculation in Gaussian03 [14]. Histidine residues were assumed to be charged, with the ND and NE atoms protonated; Arginine and Lysine residues were assumed to be protonated. All systems were solvated using explicit TIP3P water model [15], in a cubic box with a margin of 10 Å and neutralized by adding Sodium counter-ions. The Particle-Mesh Ewald method was applied to calculate long-range electrostatic interactions with a cutoff distance range of 10 Å, [16] and a Lennard-Jones 6–12 potential was used to evaluate van-der-Waals interactions within the cut-off range of 10 Å. The LINCS algorithm of fourth order expansion was used to constrain bond lengths [17]. After solvation and neutralization, each system was energy minimized for 10,000 steps using steepest-descent optimization method to remove poor van-der-Waals contacts in the initial geometry. After minimization, two stages of equilibration were conducted. First, the system was equilibrated for 100 ps with position restraints of 10,000 kJ/mol on all heavy atoms. A constant temperature of 300 K was maintained using the V-rescale algorithm [18], with a coupling time of 0.1 ps and separate baths for the solute and the solvent. Second, the pressure was kept constant at 1 bar using the Parrinello–Rahman pressure coupling scheme with a time constant of 2 ps [19]. Initial velocities were generated randomly using a Maxwell–Boltzmann distribution corresponding to 300 K. Neighbor lists were updated every 10 fs using a group cut-off scheme. Finally, the production run was performed for 100 ns without restraints at 300 K in the isothermal-isobaric ensemble.

Interaction analysis was performed using g_dist tool in the GROMACS package to measure the inter-atomic distances. The g_rms tool of the GROMACS package was used to calculate the root mean square deviations (RMSD) during the trajectories taking the minimized crystal structure as a reference. Root mean square fluctuations (RMSF) of the backbone of each residue were calculated by g_rmsf while g_mmpbsa tool is used to calculate the binding energy.

## Results

### DST and spoligotypes

The isolates had a variety of characteristics in terms of drug resistance to the drugs (SIRE/SHRE) with ofloxacin and spoligotypes as elaborated in Table 1. As obtained in Table 1, isolates used in the study ranged from pan-susceptible to complete resistance towards the tested drugs. The same stands true for their spoligotypes, reflecting different dominances in different geographical locations with more of Beijing strains in recent isolates.

### Sequencing results

No mutation, except in one, could be observed in the region sequenced. A single mutation that was found was G to A resulting in the change of amino acid from Valine (GTC) to Isoleucine (ATC) in isolate N16. The isolate showed a change in one nucleotide as compared with wild-type sequence at the 664th position from the start of the codon i.e. A (TG….); this corresponds to the position 16,807 of the template. The sequence was submitted to NCBI and have Genbank accession number as KX029321. This change is reported as Non-synonymous SNP in Polytb (http://pathogenseq.lshtm.ac.uk/polytb) and GMTV (http://mtb.dobzhanskycenter.org/) SNP databases. However, the isolation sites were other than India, spoligotypes were different and no comment on its impact on protein structure or function could be found.

### MD simulations results

The conformation achieved from 100 ns of simulation is more stable and credible than docked conformations. This can be justified by the fact that docking methods have some inherent pitfalls and approximations. However, MD-simulations represent more realistic conditions with the physiological environment. Therefore, the binding orientation of Mitoxantrone (ligand) predicted through MD-simulations shows better correlation to their biologically active state. The structural and dynamical property of the complex has been analyzed through the trajectories obtained from the MD-simulations. The trajectory data of complex were plotted for energy, RMSD, and RMSF and the interaction analysis of the ligand have been done within the binding pocket.

**Fig. 1:**
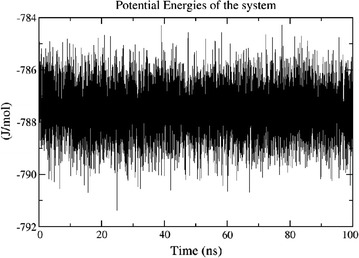
Energy plot of MD simulations for protein–ligand (Mitoxantrone) complex as a function of time

**Fig. 2:**
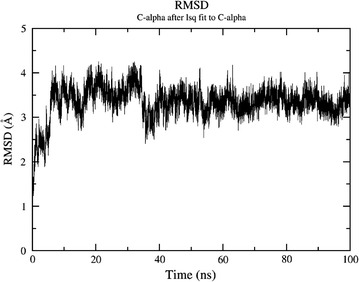
Root mean square deviation (RMSD) of Protein of C-alpha atoms with respect to the time over the course of 100 ns of MD simulation

**Fig. 3:**
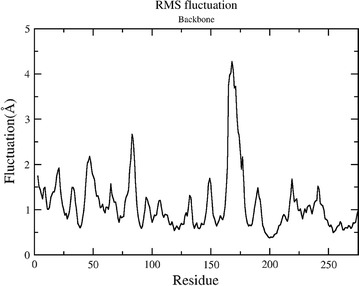
Root mean square fluctuation (RMSF) of residue backbone with respect to the average structure during the course of 100 ns of MD simulations

**Fig. 4:**
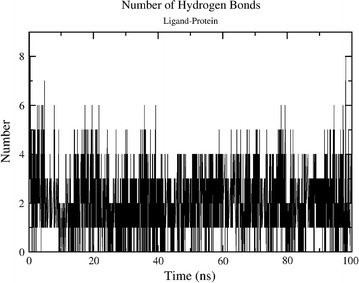
Average number of Hydrogen bond between ligand and protein during the entire 100 ns of simulations

The potential energy of the system is quite stable throughout the simulations as shown in Fig. 1. The trajectories reveal that secondary structure of the protein is maintained throughout the simulation. RMSD plot in Fig. 2 shows that after an initial equilibration period of about 25 ns the system reached a plateau and the RMSD value never crossed the mark of 3.5 Å from the initial structure in the entire simulation of 100 ns. The flexibility of the structure is measured in terms of RMSF. Figure 3 demonstrates the flexibility of backbone with respect to an average structure which is below 2 Å. Only for loops it is a little bit higher and reaches a maximum of 3 Å. The interactions which are responsible for keeping the ligand inside the pocket are analyzed by a number of H-bond interactions. Figure 4 reveals that average 3 H-bonds between 2FUM protein and the ligand, Mitoxantrone, is quite persistent and sometimes 4 H-bonds were also seen which reached a maximum of 6–8. Further, we analyzed the particular residue responsible for these interactions as well.

### Interaction analysis

**Fig. 5:**
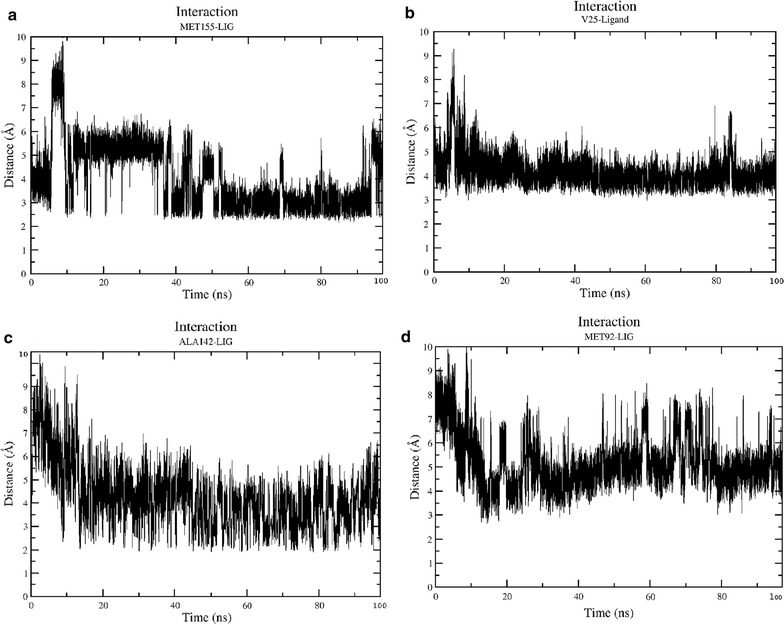
Interaction of residues with the ligand. **a** Interaction of Methionine-155 with the ligand. **b** Interaction of Valine-25 with the ligand. **c** Interaction of Alanine-142 with the ligand **d** Interaction of Methionine-142 with the ligand

The persistence of interaction is quantified by distance analysis between particular residues in the binding pocket with the specific atoms of Mitoxantrone. Figure 5 reveals the most persistent interaction found in simulations of 100 ns. Figure 5a shows the interaction of MET-155 with the ligand in which after initial equilibration period of 40 ns the interaction is quite stable and distance is below 3 Å while with VAL-25 it is quite persistent (about 3.75 Å) from the beginning of the simulation. The most surprising interaction found with ALA-142 with the ligand. Figure 5c shows that initially residue was distant (~7 Å apart) but after equilibration time the conformation of protein switched in a manner that ALA-142 started interacting with the ligand. Similarly, Fig. 5d also shows that initially the residue MET-92 was distant from the ligand but after equilibration time the conformation of protein switched in a manner that MET-92 started interacting with the ligand. These interactions were the major binding source for the ligand in the binding cavity.

### MMPBSA results

**Fig. 6:**
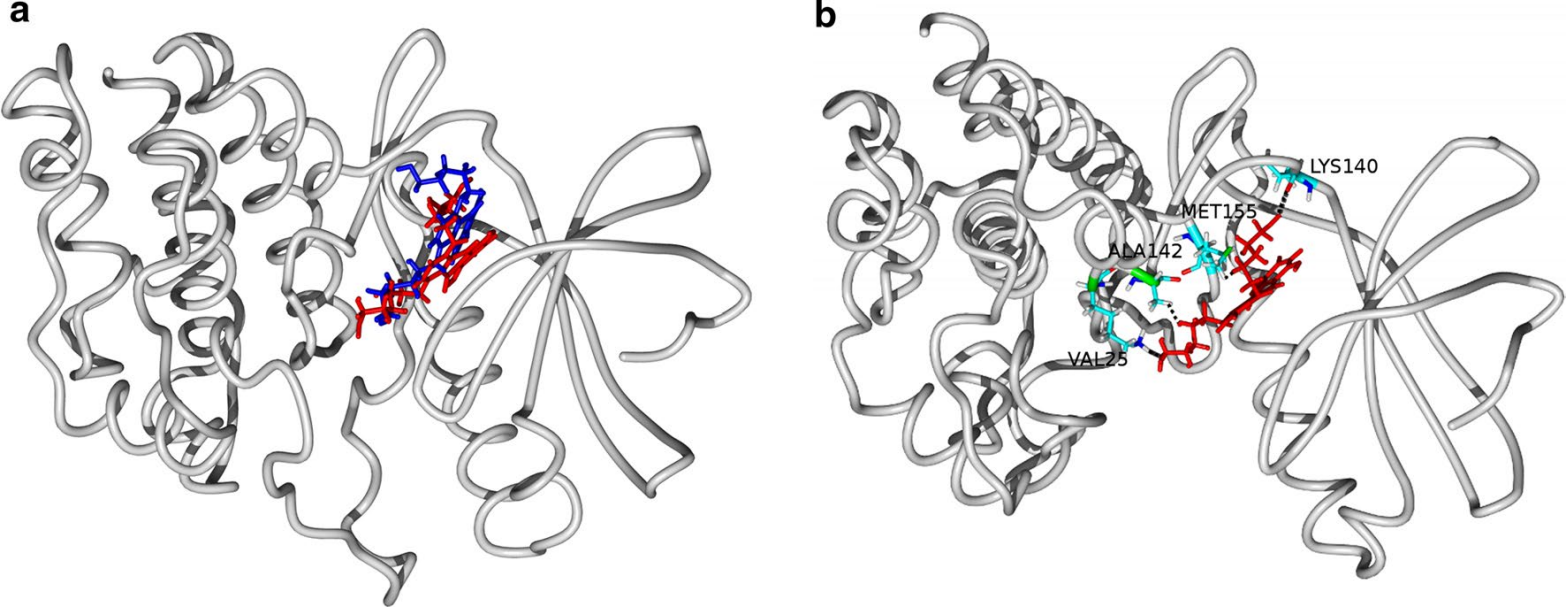
The orientation of Mitoxantrone before simulation in blue while after a simulation in red. Interaction of residue shown with side chains. Protein is represented in tube (grey colour) in both the figures (**a**, **b**)

The binding energy of the ligand is calculated by MMPBSA method which is allowing the calculation of non-bonded interactions i.e., Van-der-Waal and the electrostatic terms between two sets of atoms along the length of trajectory. Each snapshot from every nanosecond was taken for our calculations. The method also allowed the calculation of polar solvation energy and SASA energy as well. In our calculation, the overall binding energy of ligand with the protein is −71.564 kJ/mol, where negative term confirms substantial affinity of the receptor for the ligand. It also confirms our molecular dynamics simulation results. In residue-wise contribution in the binding, MET-155 plays a key role and shows maximum contribution. Our interaction analysis shows persistent interaction with the ligand (Table 2). This critical residue may provide guidance for the rational design to discover a more potent drug (Fig. 6).

**Table 2:** MMPBSA calculation results

Energetic contributions	KJ/mol
Van-der-Waal energy	−225.117 ± 11.889
Electrostatic energy	−93.523 ± 15.703
Polar solvation energy	268.278 ± 23.834
SASA energy	−21.201 ± 0.721
Binding energy	−71.564 ± 19.084

## Discussion

The *M.tb* genome encodes 11 serine/threonine protein kinases (STPK) which offer attractive targets for inhibitor development [20]. PknB is part of the 11 eukaryotic-like serine–threonine protein kinases listed as an essential protein in the *M.tb* TraSH and NGS datasets [21]. The necessity of *pknB* was verified by gene knockdown experiments, which showed a remarkable decrease in bacterial viability in liquid culture upon depletion of the kinase [22]. According to Ortega and co-workers, *pknB* provides a highly potential drug target against *Mtb* as it controls both the active replicating and latent nonreplicating forms of *Mtb* [4]. They tested the role of serine/threonine protein kinases—signaling molecules and found that kinase depletion led to decrease in bacterial viability and they specifically identified the PknB kinase as a critical regulator of the oxygen-dependent replication switch. These data show that signaling through PknB modulates the growth and replication state of *Mtb* in response to oxygen. Therefore, it can also be a valuable drug target for latent TB infection.

PknB has autophosphorylation properties [23], with a cross-phosphorylation potential of other STPKs such as PknA, and PknG [24]. Moreover, PknB seems to control major metabolic pathways directly via phosphorylation of multiple protein substrates, such as the protein regulator GarA that halts the TCA cycle [25], the KasA/B proteins involved in the biosynthesis of the essential mycolic acid [26], and the Wag31 proteins involved in cell division and morphology [27]. Likewise, virulence factors SigH and RshA [28], and cell wall biosynthetic enzymes GlmU or PBPA are also influenced [29, 30]. Thus, PknB is established as an important participant and a regulator of cell division and growth in *M. tuberculosis* proving its essentiality. However, there are no studies, to the best of our knowledge to say about the conservation status of this gene in clinical isolates with existing pressure of drugs being used for other targets.

This study tried to survey the same by sequencing the most important region of the cytoplasmic domain that participates in ligand/inhibitor binding. The crystal structure studies demonstrated that mitoxantrone is an ATP-competitive inhibitor of PknB and Leu17, Gly18, Val25, Ala38, Met 92, Glu93, Tyr94 and Val95 in the N-terminal lobe, Met145 and Met155 in the C-terminal lobe are the important residues taking part in the complex formation [31]. Moreover, we used high fidelity enzyme for amplification of *pknB* gene from genomic DNA to ensure error-free amplification before sequencing.

## Conclusions

In our study, *pknB* was confirmed to be conserved, even in clinical isolates with different drug resistance and spoligotype profile. This was observed in sequencing experiments with 116 clinical isolates having susceptibility profiles ranging from multidrug resistant isolates with resistance to individual drugs including fluoroquinolones to the ones with complete susceptibility towards tested drugs. The choice of clinical strains of *M.tb* to probe the conservation pattern of the target region is the best strategy because of different drug exposures to them along with host’s immune pressure. Such isolates are under pressure to acquire drug resistance by the modification of the drug targets. Bioinformatics studies using hypothetical mutations in binding sites were also done to access the mutations which may disturb the drug binding and induce resistance. The absence of mutations in overall sequence including putative drug binding domains proved that pknB remain a good target for drug development. Even the highly resistant isolates did not have any mutations in this region. This also reveals that sequences in *pknB* are not influenced by the change in other genomic regions. However, a study is required to see if constant usage of inhibitors of this particular protein exerts a selection pressure and how susceptible is the target for mutations under specific drug pressure.
